# Trübung der Intraokularlinse nach kombinierter Katarakt- und minimal-invasiver Glaukomchirurgie (MIGS)

**DOI:** 10.1007/s00347-020-01176-5

**Published:** 2020-07-17

**Authors:** Christoph Kern, Siegfried Priglinger, Marc J. Mackert

**Affiliations:** Augenklinik der Universität München, Ludwig-Maximilians-Universität, Mathildenstr. 8, 80336 München, Deutschland

**Keywords:** MIGS, Microshunt, Nd YAG Laser, IOL-Trübung, Fibrinreaktion, MIGS, Microshunt, Nd YAG laser, IOL opacification, Fibrinous inflammation

## Abstract

Drei Wochen nach komplikationsloser kombinierter minimal-invasiver Glaukom- und Kataraktoperation kam es zu einer Sehverschlechterung durch die Eintrübung der Intraokularlinse. Durch die Nichtanwendung der postoperativen antiinflammatorischen Lokaltherapie kam es zu einer homogenen Fibrinbildung auf der Linsenvorderfläche sowie einer beginnenden Vernarbung des Sickerkissens. Wir führten ein Needling mit 5‑Fluorouracil sowie eine Linsenpolitur mit dem Nd:YAG-Laser durch und erreichten so eine suffiziente Druckkontrolle und Sehverbesserung.

## Abstract (en)

A visual deterioration due to opacification of the intraocular lens was observed 3 weeks after uncomplicated combined minimally invasive glaucoma and cataract surgery. Due to the patient’s failure to apply the prescribed postoperative local anti-inflammatory eyedrops, homogeneous fibrin coating on the anterior surface of the lens occurred, as well as early scarring of the filtering bleb. We performed needling with 5‑fluorouracil and lens polishing with the Nd:YAG laser to achieve sufficient pressure control and vision improvement.

Seit 2019 ist der Microshunt als Verfahren der minimal-invasiven Glaukomchirurgie (MIGS) zugelassen und kann als Stand-alone-Verfahren oder in Kombination mit einer Kataraktoperation implantiert werden.

## Anamnese

**Figure 1:**
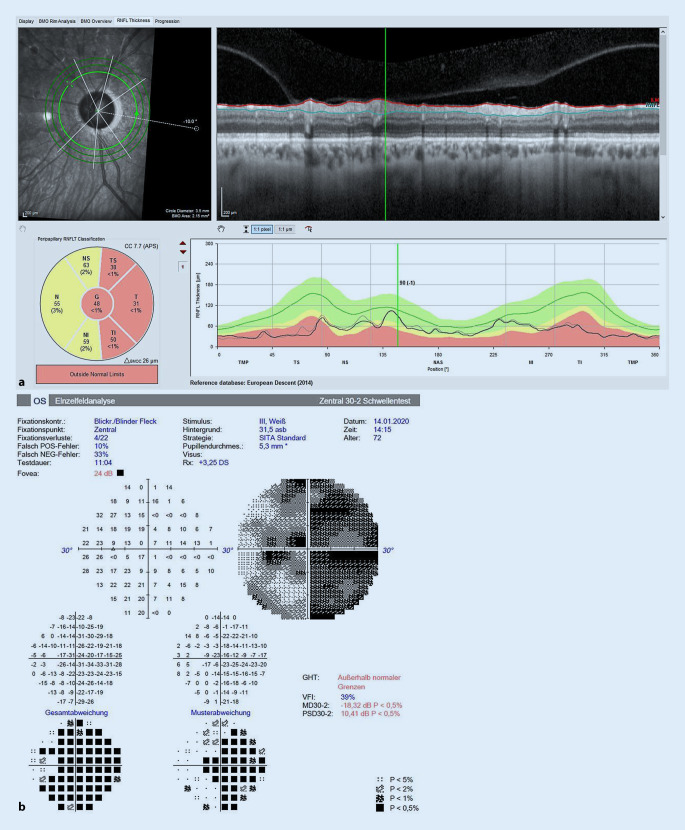

Eine 72-jährige Patientin wurde bei fortgeschrittener Linsentrübung und glaukomatöser Optikusatrophie bei primärem Offenwinkelglaukom (POWG) zur weiteren Behandlung an unsere Klinik überwiesen. In der Nervenfaserschichtmessung und im 30° Gesichtsfeld zeigten sich linksseitig betont zusätzlich zur fortgeschrittenen Katarakt ausgeprägte glaukomatöse Veränderungen am Sehnerven und damit verbundene Gesichtsfelddefekte (Abb. 1). Wir entschieden uns für ein kombiniertes Vorgehen im Rahmen einer minimal-invasiven Glaukomchirurgie (MIGS) und führten eine Kataraktextraktion mit Implantation einer Intraokularlinse (IOL; enVista MX60P, Bausch & Lomb, Rochester, NY, USA) in Kombination mit der Implantation eines Microshunts (Preserflo, Santen, Osaka, Japan) zur Senkung des Augeninnendrucks am linken Auge durch. Intraoperativ erfolgte die subkonjunktivale Applikation von Mitomycin C (0,2 mg/ml). Präoperativ lag die bestkorrigierte Sehschärfe (BCVA) bei 0,3 dezimal und der Augeninnendruck (IOD) applanatorisch gemessen (Pachymetrie 546 µm) bei 19 mm Hg unter lokaler Maximaltherapie. Der kombinierte Eingriff wurde komplikationslos durchgeführt, und die Patientin wurde nach subkonjunktivaler Gabe von 5‑Fuorouracil am zweiten postoperativen Tag und einem Augeninnendruck von 8 mm Hg in die ambulante Betreuung entlassen. Die auf Station begonnene stündliche Eingabe von steroidhaltigen Augentropfen (1 % Prednisolonacetat) sollte nach Entlassung von der Patientin fortgeführt werden. Drei Wochen nach komplikationsloser Operation stellte sich die Patientin in unserer Klinik wieder vor und klagte über eine Sehverschlechterung am operierten linken Auge. In der ersten Woche nach der Operation sei das Sehen sehr gut gewesen.

## Befund

**Figure 2:**
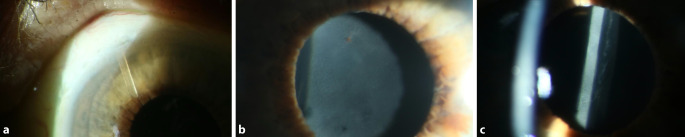

Bei Wiedervorstellung lagen die BCVA am betroffenen Auge bei 1/35 Metervisus und der IOD bei 22 mm Hg. Der Microshunt lag bei 11 Uhr in der Vorderkammer in loco, sodass ein regelrechter Abfluss gewährleistet war. Das Sickerkissen war flach und zeigte erste Hinweise für eine beginnende Vernarbung. Die IOL war regelrecht im Kapselsack implantiert. Es zeigte sich jedoch eine anteriore Trübung des Linsenmaterials (Abb. 2).

## Diagnose

Bei genauerer Betrachtung ist bei 12 Uhr eine Pigmentauflagerung auf der Trübung erkennbar, was für das Vorliegen einer gelösten posterioren Synechierung spricht, wie sie bei intraokularen fibrinösen Entzündungen vorkommt (Abb. 2b). Bei 6 Uhr zeigte sich zudem eine kleine punktuelle Öffnung, hinter der eine klare IOL erkennbar ist (Abb. 2b). Diese Befunde machen die Verdachtsdiagnose einer frühen Eintrübung des Linsenmaterials eher unwahrscheinlich, sodass wir im weiteren Verlauf von einer homogenen Fibrinauflagerung im Sinne eines anterioren Fibrin-Coatings der IOL ausgingen. Die Erhöhung des IOD erklärte sich über das flache Sickerkissen mit bedingter beginnender Vernarbung durch die protrahierte Entzündungsreaktion.

## Therapie und Verlauf

**Figure 3:**
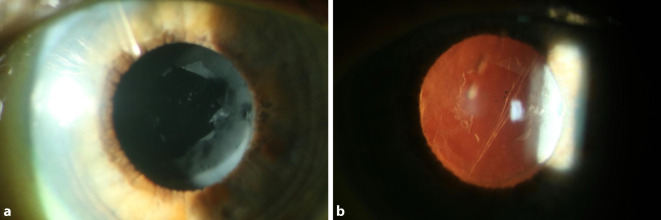

Bei insuffizienter Drucksenkung führten wir zur Verbesserung des Kammerwasserabflusses ein Needling des Sickerkissens mit 5‑Fluorouracil zur Fibrosehemmung durch, woraufhin sich der IOD bei den Folgeuntersuchungen auf 8–10 mm Hg ohne drucksenkende Medikation stabilisierte. Einige Tage nach der Sickerkissenrevision und intensiver lokaler Steroidtherapie mit 1 % Prednisolonacetat-Augentropfen wurde eine anteriore Linsenpolitur mit einem Neodym-dotierten Yttrium-Aluminium-Granat-Laser (Nd:YAG-Laser) durchgeführt (Ultra Q Reflex, Ellex, Medical Lasers Ltd., Awson Lakes, Australien). Insgesamt wurden 100 Schüsse mit einer Energie von 0,6–0,8 mJ unter Verwendung eines Kapsulotomiekontaktglases appliziert, wodurch sich eine kreisrunde Öffnung von 3,5 mm Durchmesser in der Fibrinauflagerung schaffen ließ (Abb. 3). Schon am Tag nach dem Eingriff bemerkte die Patientin eine subjektive Sehverbesserung, und die BCVA stieg auf 0,32 dezimal an. Bei kleiner Öffnung der Fibrinmembran auf der Linsenvorderfläche erweiterten wir die YAG-Laserung im Verlauf, um Blendphänomene zu vermeiden.

## Diskussion

Die minimal-invasive Glaukomchirurgie, kurz MIGS genannt, ist aufgrund effizienter Drucksenkung und guten Sicherheitsprofils in vielen Fällen eine gute Alternative zur Trabekulektomie (TET) [3]. Das Microshunt-Implantat lässt sich im Gegensatz zur TET in Kombination mit einer Kataraktoperation ab externo implantieren, ohne dass ein nachteiliger Effekt entsteht [2, 7]. Jedoch ist auch bei minimal-invasiven Verfahren die postoperative Entzündungsprophylaxe mit Steroiden von entscheidender Bedeutung für den Erfolg der Operation, im Speziellen bei kombinierten Verfahren [9]. Ein Needling ist bei bis zu 9 % der Fälle nach kombinierter Operation mit Implantation eines subkonjunktivalen Mikroshunts nötig, um eine suffiziente Drucksenkung zu erreichen [6]. Dies war auch im vorgestellten Fall notwendig.

Trübungen der IOL sind für alle Linsenmaterialen beschrieben, jedoch sind hydrophile Acrylat-Linsen anfälliger für Materialtrübungen; diese entstehen infolge von Kalziumablagerungen auf der Linsenvorderfläche insbesondere nach Vitrektomie mit Gaseingabe, aber auch durch eine intrakamerale Entzündung, den damit verbundenen Zusammenbruch der Blut-Kammerwasser-Schranke mit erhöhter Konzentration von Kalzium in der Vorderkammer [4, 8]. In unserem Fall erfolgte die Implantation der hydrophoben Acrylat-Linse (enVista MX60P) komplikationslos, und es gibt bisher keine Fallberichte einer Materialtrübung dieses Linsentyps. Auf Nachfrage gab unsere Patientin an, seit ihrer Entlassung keine steroidhaltigen Augentropfen appliziert zu haben. Ein postoperativer Vorderkammerreiz durch mangelnde Applikation steroidhaltiger Augentropfen kann also zu einer Ablagerung von Kalziumkristallen oder Fibrin auf der Linsenvorderfläche geführt haben. Bei genauer Betrachtung zeigte sich eine punktuelle Pigmentauflagerung auf der Linsentrübung im Sinne einer gelösten anterioren Synechierung, sodass wir von einer Fibrinauflagerung ausgehen mussten (Abb. 2). Generell könnten Materialtrübungen der IOL mit Fibrinablagerungen auf der Linsenvorderfläche verwechselt werden, was das Phänomen der reversiblen IOL-Trübungen (nach Resorption des Fibrins) erklären würde.

In der Literatur ist die Bildung von Fibrin nach kombinierter Kataraktoperation und MIGS vorbeschrieben: So kam es am ersten postoperativen Tag bei einer 72-jährigen Patientin zu einer Okklusion des Shunt-Lumens, die erfolgreich mittels Nd:YAG-Laser behandelt wurde [5]. Infolge dieser Beobachtungen wird nach MIGS, insbesondere nach kombinierter Operation, zu einer intensiven topischen antiinflammatorischen Therapie geraten [9]. Auch eine intrakamerale Gabe von rekombinantem Gewebe-Plasminogen-Aktivator (rTPA) wäre bei unserer Patientin infrage gekommen. In Fallberichten hat rTPA eine gute Wirkung auf die fibrinösen Membranen, kann jedoch selbst Materialtrübungen der IOL induzieren [1]. Aus diesem Grund entschieden wir uns gegen eine Vorderkammerspülung mit rTPA-Eingabe während des Needlings und führten einige Tage später die Behandlung mit dem Nd:YAG-Laser durch. Zudem bestand initial die Hoffnung, dass eine intensive Lokaltherapie mit steroidhaltigen Augentropfen zu einer Resorption des Fibrins führt.

Eine Kombination aus MIGS und Kataraktchirurgie stellt eine sichere und effiziente Methode zur Drucksenkung und Visusrehabilitation dar. Insbesondere im postoperativen Verlauf muss auf eine intensive antiinflammatorische Therapie geachtet werden, da sonst Komplikationen wie eine frühe Vernarbung des Sickerkissens oder eine intraokulare Fibrinreaktion auftreten können.

## Fazit für die Praxis

- Bei gutem Sicherheitsprofil kann die MIGS als Option zur klassischen filtrierenden Glaukomoperation bei Patienten in Kombination mit einer Kataraktoperation erwogen werden.
- Eine intensive Anwendung topischer Steroide im postoperativen Verlauf ist auch bei MIGS, insbesondere bei kombinierten Eingriffen, zur Vermeidung einer postoperativen intraokularen Entzündung unumgänglich.
- Bei früher Eintrübung des IOL-Materials auch an Fibrin-Coating denken.
